# Translational profiling in childhood acute lymphoblastic leukemia: no evidence for glucocorticoid regulation of mRNA translation

**DOI:** 10.1186/1471-2164-14-844

**Published:** 2013-12-01

**Authors:** Tatsiana Aneichyk, Daniel Bindreither, Christine Mantinger, Daniela Grazio, Katrin Goetsch, Reinhard Kofler, Johannes Rainer

**Affiliations:** Division of Molecular Pathophysiology, Biocenter, Innsbruck Medical University, Innsbruck, 6020 Austria; Tyrolean Cancer Research Institute, Innsbruck, 6020 Austria

**Keywords:** Polysome profiling, Translatome, Gene regulation, Glucocorticoids, Acute lymphoblastic leukemia

## Abstract

**Background:**

Glucocorticoids (GCs) are natural stress induced steroid hormones causing cell cycle arrest and cell death in lymphoid tissues. Therefore they are the central component in the treatment of lymphoid malignancies, in particular childhood acute lymphoblastic leukemia (chALL). GCs act mainly *via* regulating gene transcription, which has been intensively studied by us and others. GC control of mRNA translation has also been reported but has never been assessed systematically. In this study we investigate the effect of GCs on mRNA translation on a genome-wide scale.

**Results:**

Childhood T- (CCRF-CEM) and precursor B-ALL (NALM6) cells were exposed to GCs and subjected to “translational profiling”, a technique combining sucrose-gradient fractionation followed by Affymetrix Exon microarray analysis of mRNA from different fractions, to assess the translational efficiency of the expressed genes. Analysis of GC regulation in ribosome-bound fractions *versus* transcriptional regulation revealed no significant differences, i.e., GC did not entail a significant shift between ribosomal bound and unbound mRNAs.

**Conclusions:**

In the present study we analyzed for the first time possible effects of GC on the translational efficiency of expressed genes in two chALL model systems employing whole genome polysome profiling. Our results did not reveal significant differences in translational efficiency of expressed genes thereby arguing against a potential widespread regulatory effect of GCs on translation at least in the investigated *in vitro* systems.

**Electronic supplementary material:**

The online version of this article (doi:10.1186/1471-2164-14-844) contains supplementary material, which is available to authorized users.

## Background

Glucocorticoids (GCs) are natural stress-induced steroid hormones, synthesized and secreted by the adrenal cortex. GC plays an important role in the regulation of glucose metabolism and is part of the anti-inflammatory feedback mechanisms of the immune system. In addition to many other immunological and metabolic effects, GC causes cell cycle arrest and cell death in lymphoid cells, an effect that led to the use of GC in essentially all chemotherapy protocols for lymphoid malignancies, particularly childhood acute lymphoblastic leukemia (chALL) [1]. Unfortunately, GC therapy is not equally effective in all patients. The degree of the early response to GC treatment is a determinant of the intensity of subsequent therapy, and is also a prognostic factor of the overall outcome. Therefore, further investigation of molecular mechanisms responsible for GC-induced apoptosis is required to better understand the phenomenon of GC resistance.

It is generally believed that GC exerts most of its effects through the GC receptor (GR, NR3C1), a ligand-activated transcription factor of the nuclear hormone receptor superfamily. In the absence of ligand, GR resides in the cytoplasm in an inactive multi-protein complex consisting of two hsp90 molecules and a number of other proteins, including the immunophilins p59 and calreticulin [2]. Upon ligand binding, the GR undergoes a conformational change that causes dissociation of the multi-protein complex. GRs can then form a dimer that translocates into the nucleus, where it transcriptionally activates response genes, generally by binding specific DNA sequences known as glucocorticoid response elements (GRE). GRs can also remain monomers and repress the activity of some transcription factors [3].

Microarray technology allows analysis of gene expression on a genome-wide scale, and thus has been extensively used to identify transcriptional response genes *in vitro* in cell line systems [4–8] as well as in chALL patients, *in vivo*[9] and *ex vivo*[10]. Lists of candidate genes have been generated, some of which have been functionally investigated [11], but the exact mechanisms of action of GCs remain to be elucidated. In addition to the widely studied transcriptional regulation, some studies suggested translational regulation by GCs of selected genes [12–14], but this question has never been addressed systematically.

“Translational profiling” is a technique that combines polysome profiling using sucrose gradient fractionation with microarray technology to estimate translational efficiency on a genome-wide scale. This technique, although not directly measuring translation *per se*, is widely accepted to estimate the translational efficiency of mRNAs [15, 16] and has been shown to provide a useful estimate of protein synthesis [17].

In this project, we performed a genome-wide investigation of translational regulation by GC in chALL. Comparison of GC regulations in ribosome-bound fractions against gene regulations in the full data set (i.e., transcriptional gene regulations) revealed no significant difference, thus suggesting that GCs do not influence mRNA translation, at least in the cell systems analyzed. In addition, our data enables evaluation of the translational efficiency of genes expressed in the investigated systems.

## Results and discussion

**Figure 1:**
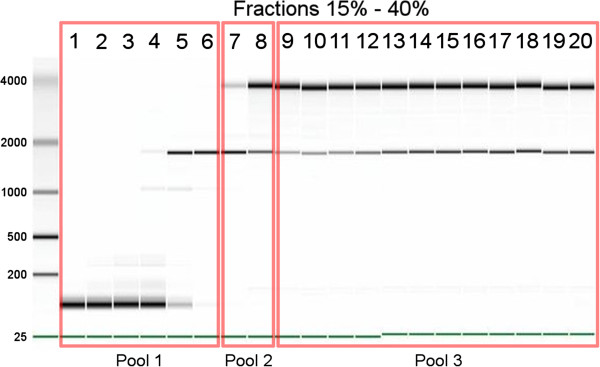
**Representative example of Agilent gel electrophoresis of RNA fractions obtained by sucrose gradient separation from NALM6 cells.** Fractions 1–6 (pool 1) encompass non-ribosome bound RNAs, as suggested by the complete absence of 28S RNA. Fractions 7–8 form an intermediate pool potentially containing translationally-initiated mRNAs (pool 2). Fractions 9–20 (pool 3) contain mRNAs bound to ribosomes, as evidenced by the presence of both ribosomal subunits 18S and 28S.

To address the question of translational regulation by GC, two different GC-sensitive cell lines, representing two major sub-types of ALL, were investigated: the CCRF-CEM cell line corresponding to the childhood T-ALL sub-type, and the NALM6 line, corresponding to pre-B ALL. Three independent experiments for each cell line were performed. Cells were cultured for 6 hours in the presence or absence of GC followed by classical sucrose gradient fractionation [18–21]. Twenty one to 23 different fractions were assigned to one of three pools based on the presence of different ribosomal subunits, referred to as non-translated (pool 1), intermediate (pool 2) and translated (pool 3) pools (see Figure 1 and Additional file 1:Figure S9). While this pooling of fractions from sucrose gradients precludes drawing conclusions on the regulation of translation elongation or termination, it allows analyzing translational regulation at the step of translation initiation. The initiation phase is in fact the rate-limiting step in the process of protein synthesis and also the most common target in translational regulation [20, 22, 23]. Samples were then hybridized to Affymetrix Exon 1.0 microarrays for gene expression profiling, resulting in total of 36 microarrays (2 cell lines, 2 treatments, 3 pools, 3 replicates).

We pre-processed the microarrays using a modified version of the GCRMA method [8], which resulted in summarized expression values per transcript probe set. These transcript probe sets were annotated to IDs, gene names, transcript biotypes and other information according to the Ensembl database version 67. A single “representative” transcript probe set was chosen for genes measured by multiple transcript probe sets on the microarray. Our selection prioritized the protein coding transcripts with high numbers of probes and highest average expression throughout arrays (see Methods for detailed description). There were a total of 41325 genes, as defined by the Ensembl database, detectable on the microarrays, each represented by a chosen transcript. For further analysis, we restricted the data to presumably expressed genes (expression level higher than 3 in at least one pool, with expression values of the microarrays ranging from 0 to 16 in log2 scale). For CEM-C7H2, 13874 genes were considered to be expressed, and for NALM6, 16289 (see Table 1).

**Table 1:** **Summary of the biotype assignment of genes expressed in the two cell lines**

	C7H2	NALM6	Detectable*:
protein_coding	10318	11129	19229
processed_transcript	236	297	1010
sence_intronic	32	55	353
misc_RNA	156	217	784
snRNA	111	176	969
miRNA	42	56	196
snoRNA	205	264	745
rRNA	51	82	276
scRNA_pseudogene	213	232	620
Mt_tRNA_presudogene	31	44	277
Other biotypes	2479	3737	16866
Total number of genes:	13874	16289	41325

Summary of the biotype assignment of genes expressed in the two cell lines and of all genes detectable on the microarray. Other biotypes include pseudogene, lincRNA, antisense, polymorphic_pseudogene, non_coding, IG_V_pseudogene, ncrna_host, IG_C_gene, IG_J_gene, IG_V_gene, TR_J_gene, TR_V_gene, TR_V_pseudogene, TR_C_gene, TR_J_pseudogene, IG_C_pseudogene, IG_D_gene, sense_overlapping, 3prime_overlapping_ncrna, snoRNA_pseudogene, rRNA_pseudogene, tRNA_pseudogene, snRNA_pseudogene, miRNA_pseudogene, misc_RNA_pseudogene. Total number of genes annotated by Ensembl version 67: 51455. *) Detectable on the microarray.

**Figure 2:**
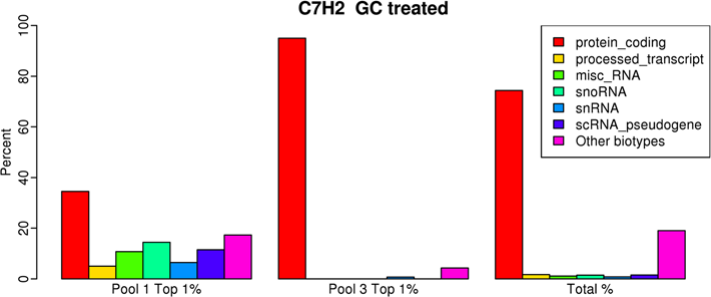
**Distribution of biotypes for the genes with the highest relative expressions in the corresponding pool.** Distribution of biotypes in pools 1 (left panel) and 3 (middle panel) for the top 1% of genes with the highest relative expression in the corresponding pool compared to the total number of genes on the microarray (right panel). Shown are the results for EtOH-treated C7H2 cells. For the complete list of biotypes of the genes detectable on the microarray, see Table 1.

To estimate the translational efficiency of the transcripts, we calculated the relative expression (RE) in each pool using a measure representing the percentage of RNA in each of the three pools for the respective gene. We will refer to RE in pool 3 as translational efficiency of the gene (see Methods) following the definition in [17]. We next selected the top 1% of genes with the highest RE in different pools and grouped them according to transcript biotypes, a classification scheme defined in the Ensembl database that groups genes or transcripts based on their function or structure. There were 36 different biotypes, including protein coding, pseudogene, lincRNA etc. The full list of biotypes represented on the microarray, as well as the number of genes of each biotype, are shown in Table 1. Figure 2 shows the presence of different biotypes among the top 1% of genes with the highest RE in corresponding pools for the CEM-C7H2 cell line treated with GC. We observed an enrichment of non-coding biotypes among those with the highest RE in pool 1, consistent with the assumption that this pool contained non-translated RNA. Similarly, among genes with the highest RE in pool 3, > 95% were protein coding. Both cell lines and both treatments showed very similar results (Additional file 1: Figure S1).

**Figure 3:**
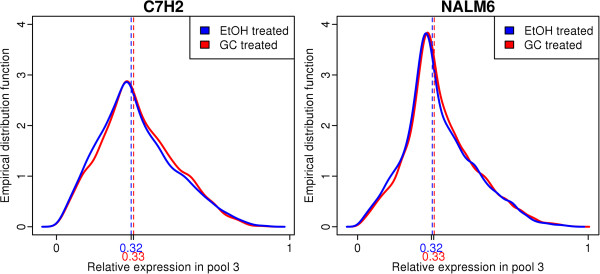
**Distributions of translational efficiencies of protein-coding genes.** Distributions of translational efficiencies of all protein-coding genes expressed as proportions of the gene mRNA in pool 3 for CEM-C7H2 (left panel) and NALM6 (right panel) cells. Red and blue color corresponds to GC- and EtOH-treated samples, respectively. Dotted lines indicate the median values.

**Figure 4:**
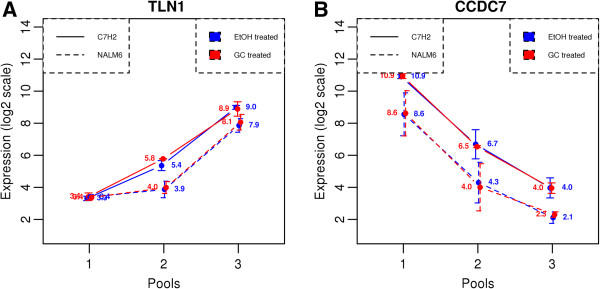
**Expression levels of mRNA for representative genes with high (A) and low (B) translational efficiencies.** X-axis indicates 3 pools: non-translated (pool 1), intermediate (pool 2) and ribosome-bound (pool 3). Y-axis represents the expression level in log2 scale. Each dot corresponds to the average expression in 3 biological experiments, error bars show standard deviation. Values for GC- and EtOH-treated samples are drawn in red and blue, respectively. CEM-C7H2 cell line is indicated by solid lines, and NALM6 by dashed lines.

Our subsequent analysis focused on protein coding genes, since the translation rate is only applicable to them. There were 10318 genes investigated in CEM-C7H2, and 11129 in NAML6. Exploratory analysis of the data indicated that the translational efficiency of protein coding genes varies significantly. Distribution of RE in pool 3 for protein coding genes is illustrated in Figure 3. The shape of the distribution for translational efficiency is consistent with results from other studies [15, 17] including a study using a different approach for assessing translational efficiency, i.e., relation of mRNA to quantitative proteomics data corrected for protein degradation [24]. Distribution for the NALM6 cell line was narrower around the median, while distribution for C7H2 is more widespread. Both cell lines skew slightly positive, suggesting fewer genes with very high translational efficiency. Distribution of RE for GC-treated and EtOH-treated samples are nearly the same, implying no global effect of GC on translational efficiency of the genes. As indicated by distribution density function, the majority of genes had their mRNA evenly distributed throughout pools. Some genes had extremely high translational efficiencies, with up to 97% of their mRNA being in pool 3. For example, TLN1 had an average translational efficiency of 0.898 in both cell lines, indicating that as much as 89.8% of its mRNA was located in pool 3 [95% confidence interval for translational efficiency was (0.885, 0.911), based on 3 experiments, both cell lines and both treatments; Figure 4A]. On the other hand, some genes appeared weakly translated, if at all. CCDC7, for example, was identified as a poorly-translated protein coding gene with translational efficiency as low as 0.011, indicating that only 1.1% of its mRNA is associated with ribosomes [95% confidence interval for translational efficiency was (0.006, 0.015) based on 3 experiments, both cell lines and both treatments; Figure 4B]. The translational efficiencies between C7H2 and NALM6 cells were highly comparable for genes expressed in both cell lines (Additional file 1: Figure S2). The average relative expression and average expression of all genes in the various pools is provided in Additional files 2 and 3 for C7H2 and NALM6 cells, respectively.

Having determined the translational efficiency for all genes we were now able to address whether genes with similar translational efficiency might also be functionally related. We tested this similar to Stevens et al. [24] by dividing all expressed protein coding genes into five groups based on their translational efficiency. On each subset we performed Gene Ontology (GO) analyses to analyze for enrichment in particular biological processes (Additional file 1: Tables S3–S14). The 3 gene sets with intermediate translational efficiency did not yield consistent results between the 2 cell lines, i.e., enrichment in particular processes was observed in one but not the other cell line. In contrast, in both cell lines the same GO terms were enriched in the gene set with the highest translational efficiencies. Similar to [24], these included RNA processing related terms (“mRNA splicing, via spliceosome”, “mRNA metabolic process” etc; Additional file 1: Table S3). Genes from the group with the lowest translational efficiencies were also enriched in both cell lines and concerned the GO terms “viral transcription” and translation related processes (“translation termination”, “translation elongation” and “translation initiation”; see Additional file 1: Table S4). In agreement with the latter finding, the synthesis of many mammalian proteins associated with the translation apparatus is regulated at the translational level [25] to allow for fast responses to changes in physiological conditions. Even in growing cells, the mRNA from about 30% of ribosomal proteins, most of which harboring the terminal oligopyrimidine tract (TOP) motif, has been described to be sequestered in mRNP particles and thus not being actively translated [25]. Furthermore, genes with TOP motifs (taken from Davuluri et al. [26]) had on average slightly lower translational efficiency than all genes (0.29 versus 0.34 in C7H2 and 0.23 versus 0.35 NALM6 cells with p-values for significance of this difference being 0.002 and < 0.001, respectively). The GO results for both cell lines and all groups are provided in Additional file 1: Tables S5 to S14.

Next, we assessed whether microRNAs (miRNAs) might be involved in determining the translational efficiency of genes. miRNAs are major post-transcriptional regulators of gene expression that are thought to bind to specific sites in the 3′ UTR of the target gene’s mRNA and subsequently repress protein synthesis by destabilizing mRNA and/or inhibiting translation [27]. miRNA mediated inhibition of translation initiation would lead to a decreased expression of the gene’s mRNA in ribosome bound fractions compared to the absolute mRNA abundance and thus result in a low translational efficiency of the gene. To test for such repressions we analyzed in each cell line the 3′ UTRs of the 5% of genes with the lowest translational efficiency for a significant over-representation of binding sites of certain miRNA families. Note that detection of repression *via* mRNA destabilization, which has been recently proposed to be the major pathway for miRNA mediated repression [28], would not be possible with the present translatome data, since it would lead to decreased mRNA levels in both ribosome bound and un-bound fractions. Among the genes with lowest translational efficiencies we identified, among others, a significant enrichment of miRNAs known to be upregulated in lymphoid disorders (see Additional file 1: Tables S15 and S16 for the full tables). Many of the poorly translated genes are predicted targets of miRNAs from the miRNA clusters miR-17 ~ 92, miR-106a ~ 363, miR-106b ~ 25 (namely miR-17, miR-19a, miR-19b, miR-20a, miR-93, miR-106a and miR-106b) as well as of miRNAs miR-30a, miR-30c, miR-30e, miR-181a, miR-181b and miR-26a. All of these miRNAs are known to be over-expressed in ALL cells or lymphoid malignancies [29] and are also highly expressed in the C7H2 cells used in the present study [6]. These results suggest that some of the poorly translated genes in C7H2 and NALM6 cells might indeed be under the control of some highly expressed microRNAs. More detailed information is provided in the supplement.

Finally, and most importantly, to investigate a possible effect of GC on translation, differential regulation analysis was performed. We defined the translational regulation of a gene as the difference between GC-regulation of ribosome-bound mRNA in pool 3 and its transcriptional regulation as defined by GC-regulation of the total mRNA regardless of the pool (see Methods). Translational regulation was calculated for each gene in each of the three experiments, and moderated t-tests [30] were performed. Resulting p-values were adjusted for multiple hypothesis testing using the method of Benjamini and Hochberg [31]. In CEM-C7H2, the lowest adjusted p-value was 0.079, and in NALM6, all adjusted p-values were close to 1, therefore none of the genes had translational regulation significantly different from 0 (at a false discovery rate of 5%). GC-treatment thus does not affect the translational efficiency of expressed genes in the two chALL model systems.

Summarizing, the RE scores for each gene provided as supplemental data will enable other researchers to evaluate the potential translational efficiency of any gene expressed at the transcriptional level in the ALL system. When analyzing groups of genes with similar translational efficiency we found for both cell lines enrichments in common biological functions for genes with very high or very low translational efficiencies. Furthermore, in an analysis of predicted miRNA target sites in the 3′ UTR of poorly translated genes, we found also evidence for miRNA mediated post-transcriptional gene repression. More importantly, our genome-wide translational profiling showed that GCs do not influence the translational efficiency of expressed genes in the two childhood ALL model systems investigated.

## Conclusions

It is well established that GC treatment leads to transcriptional gene regulations, a fact that has been extensively studied in various cell systems by us and others. Some studies, however, reported an influence of GCs on the translational efficiency of selected genes [12–14], most of them analyzed using cell free translation systems [12, 13]. Here we investigated for the first time the influence of GC treatment on translational efficiency of expressed genes on a genome-wide scale employing sucrose-gradient fractionation based ribosome profiling in combination with microarray analysis of mRNA from different fractions. Comparison of GC regulation in ribosome-bound fractions *versus* GC regulation in the full data set revealed no significant differences suggesting that GCs do not directly regulate mRNA translation. This, although being a negative result, represents a major finding of importance to the field, since it essentially excludes a potential widespread role of GCs in translational gene regulation, at least in the investigated systems. Whether this conclusion derived from *in vitro* systems extends to the *in vivo* situations remains to be shown.

In addition, we provide a comprehensive data set with translational efficiencies and average expression of all genes detectable on Affymetrix Exon 1.0 microarrays in various pooled fractions from the translatome experiments thus enabling other researchers to evaluate translational efficiencies of their candidate genes in ALL cells.

## Methods

### Cell lines and cell culture

The preB-ALL cell line NALM6 (Acc. No. 128, DSMZ-Deutsche Sammlung von Mikroorganismen und Zellkulturen, Braunschweig, Germany) and the childhood T-ALL line CCRF-CEM-C7H2 [32] were cultured in RPMI 1640 supplemented with 10% fetal calf serum and 2 mM L-glutamine at 37°C, 5% carbon dioxide, and saturated humidity. The cells were free of mycoplasma infection, and their authenticity was verified by DNA fingerprinting as detailed previously [9, 33].

### Sucrose gradient centrifugation and RNA preparation

Fifty to 100×10^6^ CEM-C7H2 or NALM6 cells were incubated with 10^-7^ M dexamethasone or 0.1% ethanol (as carrier control) for 6 h, washed twice in PBS, resuspended in 1 ml ice cold lysis buffer (10 mM Tris–HCl, pH8.0, 140 mM NaCl, 1.5 mM MgCl_2_, 0.5% Nonidet-P40, 2 mM dithiothreitol, 500U/ml RNAsin), centrifuged at 12.000 g for 10 sec at 4°C to remove the nuclei, and the supernatant (supplemented with 20 mM dithiothreitol, 150 μg/ml cycloheximide, 665 μg/ml heparin, 1 mM phenylmethylsulfonyl fluoride, 0.5% deoxycholate) was layered onto 10 ml of al linear sucrose gradient (15-40%, supplemented with 10 mM Tris–HCl, pH 7.5, 140 mM NaCl, 1.5 mM MgCl_2_, 10 mM dithiothreitol, 100 μg/ml cycloheximide, 0.5 mg/ml heparin). After 120 min centrifugation at 240.000 g at 4°C, 500 μl fractions were collected. SDS and EDTA was added to a final concentration of 1% and 10 mM, respectively, and the mixture was digested with 100 μg protein kinase K for 30 min at 37°C. Thereafter, total RNA was prepared using the Trizol method, quantiated by OD measuring at 230, 260 and 280 nm and analyzed on an Agilent Bioanalyzer (Figure 1 and Additional file 1: Figure S9). Based on the distribution of 18S and 28S RNA, the individual RNAs were combined in 3 pools, subjected to a DNase digest, purified using RNeasy MiniElute Cleanup Kit and precipitated in 2.5 M LiCl, resuspended in nuclease free water and analyzed by OD measurements and Agilent Bioanalyzer as described above.

### Microarray analysis

Affymetrix Exon 1.0 microarrays for 30 of the 36 samples were generated as described [8]. In brief, 1.5 μg of cytoplasmic RNA extracted from the sucrose gradients was depleted of rRNA and transcribed into cDNA using T7-promoter-tagged hexamer primers according to the manufacturer’s protocols and reagents. Antisense RNA was produced by T7-polymerase and subsequently transcribed into cDNA using random priming in the presence of dUTP, used to enzymatically fragment the cDNA. For the remaining 6 samples from one experiment of the NALM6 cell line, Affymetrix Exon 1.0 microarrays were generated using protocols and kits from Ambion that employ specific hexamer primers that do not bind to, and thus amplify, ribosomal RNA. The protocol was similar to the one described above with the exception of the unnecessary rRNA reduction step. The resulting targets for all samples were hybridized to human Exon 1.0 ST arrays. After washing and staining in an Affymetrix 450S fluidics station, the microarrays were scanned in an Affymetrix 3000 scanner and fluorescence signal intensities were recorded. Raw and preprocessed data have been deposited at the Gene Expression Omnibus (accession number: GSE48680).

### Probe alignment, custom annotation and pre-processing

For preprocessing of the microarrays, the R package “generalgcrma”, described in [8] was used. Probe sequences from the Exon microarray were aligned to the human genomic sequence (Ensembl version 67; genome assembly GRCh37) and annotated to the respective gene’s exon if their alignment was inside exon boundaries. Probes with multiple and partial alignments were excluded from the analysis. Custom “CEL definition files”, required for the analysis of the Affymetrix Exon 1.0 microarrays, were compiled defining a probe set for each transcript. Background adjustment, normalization and summarization of the microarray data was done using the GCRMA method [34]. As part of the preprocessing procedure, the data set was subset to a single “representative” transcript probe set per gene, as multi-transcript genes were also measured by multiple transcript probe sets on the microarray. In the selection of the representative probe set, preference was given to protein coding transcripts, transcripts with >3 probes and those with the highest average expression throughout the arrays. The choice of transcripts was performed separately for 2 cell lines, and thus different transcripts could be chosen for the same gene in different cell lines. All the further data manipulations were performed separately on the two cell lines. At the last step of preprocessing, the transcripts with very low expression levels (log2 expression lower then 3) in all three pools and both treatments were eliminated from further analysis.

### Relative expression

To evaluate the translational efficiency of the transcripts chosen for a particular gene, a measure of relative expression in each pool was used. This measure represents the percentage of RNA in each of three pools for the gene. As a result of the pre-processing, gene expression is expressed in log2 scale, thus RE is calculated as follows:

where E_i_ represents log2 expression of a transcript in pool i, i ∈ 1, 2, 3. RE in pool 3 is referred to as the translational efficiency of the gene.

### Analysis of differential regulation (translational regulation) by GC

To test our hypothesis of translational regulation by GC, pair-wise comparison of regulation by GC in pool 3 *vs* transcriptional regulation by GC was performed. To assess the significance of this differential regulation, a moderated t-test, implemented in R-package “limma” [30], was employed. The resulting p-values were adjusted for multiple hypothesis testing using the method of Benjamini-Hochberg [31]. Transcriptional regulation was defined by averaging gene expression throughout pools for each condition (GC- and ethanol-treated) using the following formula:

(where E_i_ represents log2 expression of a transcript in pool i, i ∈ 1, 2, 3). Regulation of total mRNA level (referred to as “transcriptional regulation”) was calculated as the difference between total mRNA in GC- and EtOH-treated samples:

### Gene ontology analysis

For Gene Ontology analysis we used the GOstats [35] package from Bioconductor. We employed a conditional test to enrich for more specific GO terms. All protein coding genes with an expression higher than 3 in at least one pool and treatment were used as background gene set for the hypergeometric testing, as suggested by [36].

### Analysis of genes with TOP motif

The database of classified 5′ UTRs from Davuluri et al. [26] has been downloaded from ftp://ftp.cshl.org/pub/science/mzhanglab/ramana and the provided GenBank identifiers have been mapped to NCBI Entrezgene IDs using annotation facilities from Bioconductor (i.e. the “org.Hs.eg.db” package). From the in total 2312 GenBank identifiers, only 1329 could be annotated the Entrezgene IDs, with 1312 being detectable on the microarray. Of these, 83 harbor a TOP motif in their UTR, and are thought to be poorly translated (class II in Davuluri et al.). A one tailed Student’s t-test has been employed to test whether genes with a TOP motif have on average a lower translational efficiency compared to all genes.

### miRNA target gene analysis

miRNA target gene predictions were extracted from the Targetscan database version 6.2 [37]. For the analysis, only predicted (conserved) targets of conserved miRNA families were considered. Similar to the GO analysis, a hypergeometric test was used to test for over-representation of miRNA target sites in the 3′ UTR of the 5% of genes with the lowest translational efficiency. The background gene set consisted of all protein coding genes with an expression higher than 3 in at least one pool that are potentially targeted by at least one miRNA (6899 and 7192 genes for C7H2 and NALM6 cells, respectively).

## Availability of supporting data

The data set supporting the results of this article is available in the Gene Expression Omnibus (accession number: GSE48680).

## Electronic supplementary material

Additional file 1: **Supplementary Figures and Tables.** (PDF 7 MB)

Additional file 2: **Average expression and relative expression in the various pools of all genes detected on the Exon microarray in C7H2 cells.** Columns "transcript_id", "gene_id", "probe_count", "gene_name", "gene_biotype" and "chromosome_name" contain annotations for the respective probe set id on the microarray. Columns "RE.p1", "RE.p2", "RE.p3" contain averaged relative expression across the 3 biological replicates of each gene in pools 1, 2 and 3 respectively. Columns "exprs.p1", "exprs.p2" and "exprs.p3" contain averaged expression of a gene across the 3 biological replicates (log2 scale). Suffix "GC" and "EtOH" indicate the treatment. (XLS 16 MB)

Additional file 3: **Average expression and relative expression in the various pools of all genes detected on the Exon microarray in NALM6 cells.** For a description of the content, see Additional file 2. (XLS 16 MB)
