# Supplementary material for: Translational profiling in childhood acute lymphoblastic leukemia: no evidence for glucocorticoid regulation of mRNA translation
Source: BMC Genomics. 2013 Dec 1;14(1):844. doi: 10.1186/1471-2164-14-844 (PMC4046653; doi:10.1186/1471-2164-14-844)
Supplement: Supplementary file 1 — Additional file 1: Supplementary Figures and Tables. (PDF 7 MB) [file 12864_2013_5542_MOESM1_ESM.pdf]

Supplementary Information for

Translational profiling in childhood acute lymphoblastic leukemia: no evidence for glucocorticoid regulation of mRNA translation.

Contents

Supplementary Figure 1. 1
Supplementary Figure 2. 1
Supplementary Table 1. 2
Supplementary Table 2. 2

GO-analysis on genes with similar translational efficiencys **3**
Enriched processes in both cell lines 3
Supplementary Table 3. 4
Supplementary Table 4. 4
Enriched processes in C7H2 cells 4
Supplementary Table 5. 5
Supplementary Table 6. 5
Supplementary Table 7. 6
Supplementary Table 8. 6
Supplementary Table 9. 6
Enriched processes in NALM6 cells 6
Supplementary Table 10. 8
Supplementary Table 11. 8
Supplementary Table 12. 8
Supplementary Table 13. 8
Supplementary Table 14. 9

Analysis of microRNA target genes **10**
Supplementary Table 15. 10
Supplementary Table 16. 11
Detailed information on some miRNA families 11

RNA fractions from sucrose gradient separation **16**
Supplementary Figure 9. 17

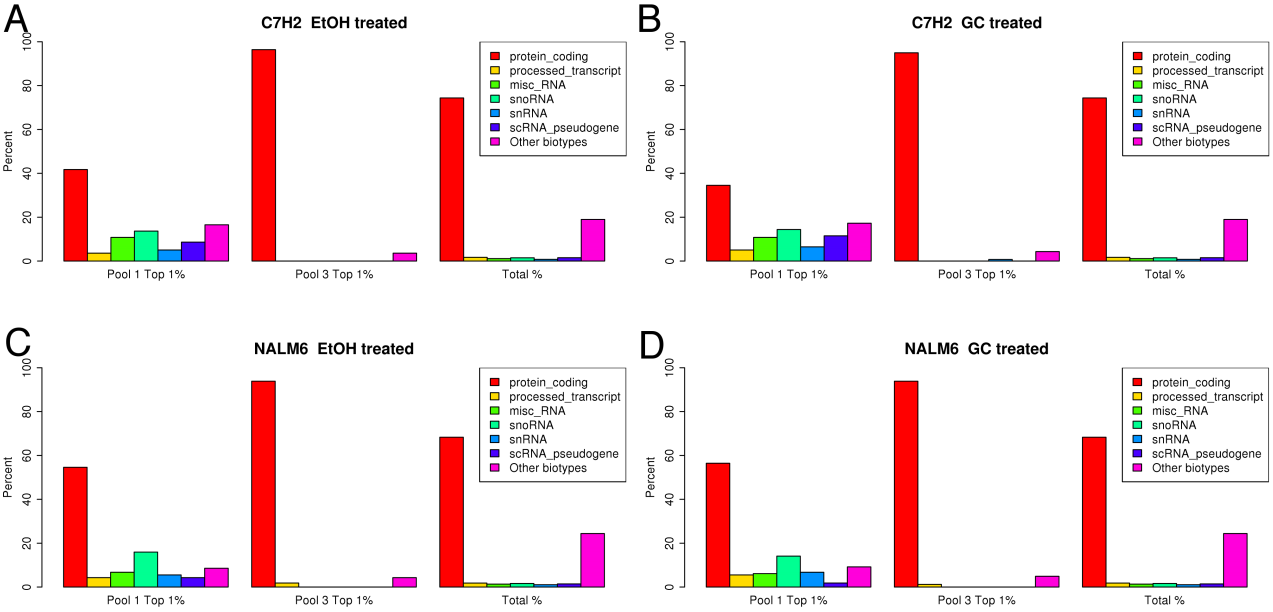

Supplementary Figure 1: Distribution of biotypes in pools 1 (left panel) and 3 (middle panel) for the top 1% of genes with the highest relative expression in the corresponding pool compared to the total number of genes on the microarray . A) CEM-C7H2 cell line, ethanol- treated, B) CEM-C7H2 cell line, GC-treated, C) NALM6 cell line, ethanol-treated, D) NALM6 cell line, GC-treated. Table 1 presents a list of all biotypes on the microarray.

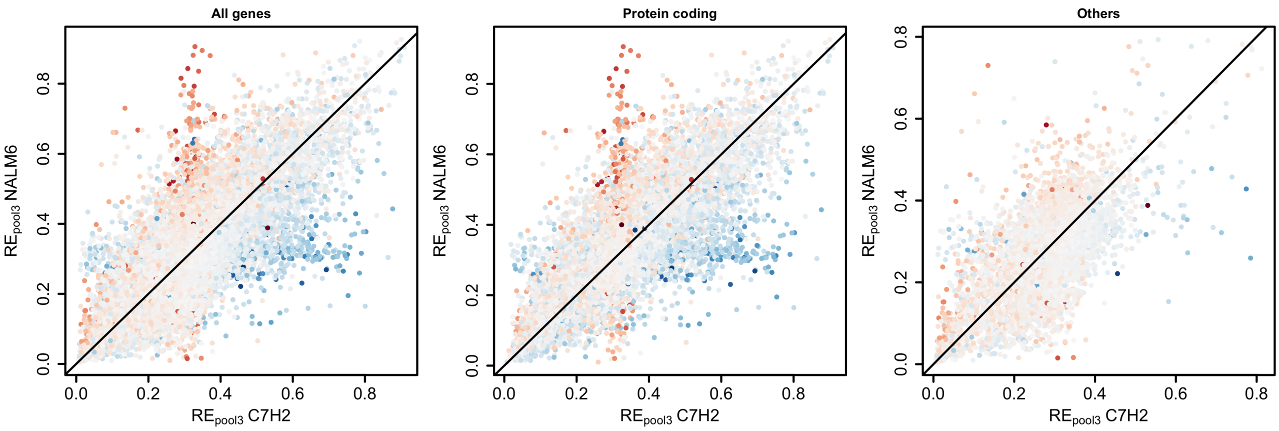

Supplementary Figure 2: Comparison of the genes' relative expressions in pool 3 in EtOH- treated control samples between C7H2 and NALM6 cells. Left: all genes detectable on the Exon microarray, middle: protein coding genes, and right: genes from all other biotypes. The colors of the data points represent the differential expression of a gene between the C7H2 and the NALM6 cell lines, with red and blue indicating higher expression in C7H2 and NALM6 cells, respectively, and white corresponding to same expression in both cell lines..

Supplementary Table 1: Average expression and relative expression in the various pools of all genes detected on the Exon microarray in C7H2 cells. Columns "transcript_id", "gene_id", "probe_count", "gene_name", "gene_biotype" and "chromosome_name" contain annotations for the respective probe set id on the microarray. Columns "RE.p1", "RE.p2", "RE.p3" contain averaged relative expression across the 3 biological replicates of each gene in pools 1, 2 and 3 respec-

tively. Columns ”exprs.p1”, ”exprs.p2” and ”exprs.p3” contain averaged expression of a gene across the 3 biological replicates (log2 scale). Suffix ”GC” and ”EtOH” indicate the treatment.

Supplementary Table 2: Average expression and relative expression in the various pools of all genes detected on the Exon microarray in NALM6 cells. For a description of the content, see Supplementary Table 1.

GO-analysis on genes with similar translational efficiency

Genes were sorted based on their translational efficiency and grouped into 5 similar-sized groups of genes. A Gene Ontology (Biological Function) analysis was performed on each of these groups using the complete set of genes as background gene set.

Enriched processes in both cell lines

	$Term$	$Size$	$Pvalue_C$	$Count_C$	$Pvalue_N$	$Count_N$
GO:0000398	mRNA splicing, via spliceosome	190	0.0000	85	0.0000	88
GO:0000375	RNA splicing, via transesterification reactions	195	0.0000	85	0.0000	88
GO:0043436	oxoacid metabolic process	577	0.0000	207	0.0000	207
GO:0016071	mRNA metabolic process	531	0.0000	175	0.0000	176
GO:0006396	RNA processing	573	0.0000	194	0.0000	182
GO:0044281	small molecule metabolic process	1226	0.0000	343	0.0000	399
GO:0022613	ribonucleoprotein complex biogenesis	215	0.0000	79	0.0000	76
GO:0046395	carboxylic acid catabolic process	119	0.0000	54	0.0000	51
GO:0002474	antigen processing and presentation of peptide antigen via MHC class I	74	0.0000	42	0.0000	34
GO:0044282	small molecule catabolic process	154	0.0000	63	0.0000	60
GO:0002478	antigen processing and presentation of exogenous peptide antigen	60	0.0000	37	0.0000	29
GO:0050878	regulation of body fluid levels	307	0.0000	106	0.0000	110
GO:0000084	S phase of mitotic cell cycle	129	0.0000	52	0.0000	50
GO:1901606	alpha-amino acid catabolic process	45	0.0000	23	0.0000	26
GO:0002479	antigen processing and presentation of exogenous peptide antigen via MHC class I, TAP-dependent	59	0.0000	36	0.0000	28
GO:0006520	cellular amino acid metabolic process	198	0.0001	67	0.0000	115
GO:0019886	antigen processing and presentation of exogenous peptide antigen via MHC class II	63	0.0000	30	0.0001	30
GO:0016192	vesicle-mediated transport	551	0.0000	162	0.0001	174
GO:0007596	blood coagulation	265	0.0000	92	0.0001	94
GO:0000216	M/G1 transition of mitotic cell cycle	73	0.0000	35	0.0001	31
GO:0044248	cellular catabolic process	1192	0.0001	318	0.0001	332
GO:0002504	antigen processing and presentation of peptide or polysaccharide antigen via MHC class II	65	0.0000	30	0.0001	30
GO:0044403	symbiosis, encompassing mutualism through parasitism	336	0.0000	112	0.0002	107
GO:0009068	aspartate family amino acid catabolic process	9	0.0000	8	0.0002	7
GO:0019048	virus-host interaction	304	0.0000	102	0.0002	97
GO:0019885	antigen processing and presentation of endogenous peptide antigen via MHC class I	6	0.0001	6	0.0001	6
GO:0019221	cytokine-mediated signaling pathway	201	0.0000	73	0.0003	69
GO:0006732	coenzyme metabolic process	136	0.0000	53	0.0004	51
GO:0034097	response to cytokine stimulus	301	0.0000	104	0.0004	96
GO:0016043	cellular component organization	2612	0.0005	642	0.0000	709
GO:0006921	cellular component disassembly involved in execution phase of apoptosis	51	0.0001	24	0.0005	23
GO:0072524	pyridine-containing compound metabolic process	34	0.0000	19	0.0006	18
GO:0000278	mitotic cell cycle	651	0.0007	179	0.0001	182
GO:0043555	regulation of translation in response to stress	13	0.0004	9	0.0004	9
GO:0048205	COPI coating of Golgi vesicle	13	0.0004	9	0.0004	9
GO:0019883	antigen processing and presentation of endogenous antigen	7	0.0007	6	0.0002	7
GO:0019882	antigen processing and presentation	79	0.0000	42	0.0009	31
GO:0051437	positive regulation of ubiquitin-protein ligase activity involved in mitotic cell cycle	67	0.0000	30	0.0009	27
GO:0033554	cellular response to stress	913	0.0002	246	0.0008	247
GO:0032057	negative regulation of translational initiation in response to stress	5	0.0006	5	0.0006	5
GO:0071702	organic substance transport	1248	0.0002	328	0.0010	336
GO:0048194	Golgi vesicle budding	14	0.0009	9	0.0009	9

Supplementary Table 3: Significantly enriched Biological Process GO-terms in both cell lines; analysis using genes with the highest translational efficiencies in each cell line. "Size": total number of genes annotated to the GO-term, "Size": number of genes from those tested for enrichment that are annotated to the GO-term. Columns with suffix "C" and "N" contain values for C7H2 and NALM6 cells, respectively.

	$Term$	$Size$	$Pvalue_C$	$Count_C$	$Pvalue_N$	$Count_N$
GO:0019083	viral transcription	130	0.0000	51	0.0000	70
GO:0006415	translational termination	78	0.0000	35	0.0000	59
GO:0006614	SRP-dependent cotranslational protein targeting to membrane	92	0.0000	36	0.0000	59
GO:0072599	establishment of protein localization to endoplasmic reticulum	94	0.0000	36	0.0000	59
GO:0006414	translational elongation	91	0.0000	35	0.0000	59
GO:0043241	protein complex disassembly	121	0.0000	43	0.0000	66
GO:0019058	viral infectious cycle	187	0.0000	59	0.0000	82
GO:0006402	mRNA catabolic process	163	0.0000	53	0.0000	78
GO:0006413	translational initiation	132	0.0000	44	0.0000	71
GO:0006612	protein targeting to membrane	129	0.0000	43	0.0000	67
GO:0000184	nuclear-transcribed mRNA catabolic process, nonsense-mediated decay	102	0.0001	35	0.0000	63
GO:0044267	cellular protein metabolic process	2303	0.0001	488	0.0000	522
GO:0033365	protein localization to organelle	398	0.0002	103	0.0000	117
GO:0044265	cellular macromolecule catabolic process	590	0.0003	143	0.0000	177

Supplementary Table 4: Significantly enriched Biological Process GO-terms in both cell lines; analysis using genes with the lowest translational efficiencies in each cell line. "Size": total number of genes annotated to the GO-term, "Size": number of genes from those tested for enrichment that are annotated to the GO-term. Columns with suffix "C" and "N" contain values for C7H2 and NALM6 cells, respectively.

Enriched processes in C7H2 cells

	Pvalue	Count	Size	Term
GO:0043436	0.000	207	577	oxoacid metabolic process
GO:0000398	0.000	85	190	mRNA splicing, via spliceosome
GO:0000375	0.000	85	195	RNA splicing, via transesterification reactions
GO:0006396	0.000	194	573	RNA processing
GO:0002478	0.000	37	60	antigen processing and presentation of exogenous peptide antigen
GO:0002474	0.000	42	74	antigen processing and presentation of peptide antigen via MHC class I
GO:0002479	0.000	36	59	antigen processing and presentation of exogenous peptide antigen via MHC class I, TAP-dependent
GO:0019882	0.000	42	79	antigen processing and presentation
GO:0016071	0.000	175	531	mRNA metabolic process
GO:0044281	0.000	343	1226	small molecule metabolic process
GO:0046395	0.000	54	119	carboxylic acid catabolic process
GO:0006099	0.000	19	26	tricarboxylic acid cycle
GO:0006950	0.000	234	804	response to stress
GO:0044282	0.000	63	154	small molecule catabolic process
GO:0051704	0.000	248	848	multi-organism process
GO:0050878	0.000	106	307	regulation of body fluid levels
GO:0034097	0.000	104	301	response to cytokine stimulus
GO:0022613	0.000	79	215	ribonucleoprotein complex biogenesis
GO:0000216	0.000	35	73	M/G1 transition of mitotic cell cycle
GO:0006977	0.000	30	59	DNA damage response, signal transduction by p53 class mediator resulting in cell cycle arrest
GO:0072474	0.000	30	59	signal transduction involved in mitotic cell cycle G1/S checkpoint
GO:0044403	0.000	112	336	symbiosis, encompassing mutualism through parasitism
GO:0007596	0.000	92	265	blood coagulation
GO:0005975	0.000	151	484	carbohydrate metabolic process
GO:0072395	0.000	30	60	signal transduction involved in cell cycle checkpoint
GO:0072422	0.000	30	60	signal transduction involved in DNA damage checkpoint
GO:0071840	0.000	42	101	cellular component organization or biogenesis
GO:0000084	0.000	52	129	S phase of mitotic cell cycle
GO:0006457	0.000	66	176	protein folding
GO:0019048	0.000	102	304	virus-host interaction
GO:0031571	0.000	31	64	mitotic cell cycle G1/S transition DNA damage checkpoint
GO:0019221	0.000	73	201	cytokine-mediated signaling pathway
GO:0031397	0.000	38	86	negative regulation of protein ubiquitination
GO:0042221	0.000	384	1430	response to chemical stimulus
GO:0006732	0.000	53	136	coenzyme metabolic process
GO:0019886	0.000	30	63	antigen processing and presentation of exogenous peptide antigen via MHC class II
GO:0051234	0.000	510	1972	establishment of localization
GO:0016032	0.000	167	560	viral reproduction
GO:0006418	0.000	24	47	tRNA aminoacylation for protein translation
GO:0006521	0.000	24	47	regulation of cellular amino acid metabolic process
GO:1901565	0.000	136	442	organonitrogen compound catabolic process
GO:0043038	0.000	25	50	amino acid activation
GO:0002504	0.000	30	65	antigen processing and presentation of peptide or polysaccharide antigen via MHC class II
GO:0034660	0.000	88	264	ncRNA metabolic process
GO:1901606	0.000	23	45	alpha-amino acid catabolic process
GO:0072524	0.000	19	34	pyridine-containing compound metabolic process
GO:0035383	0.000	25	51	thioester metabolic process

GO:0051436	0.000	29	63	negative regulation of ubiquitin-protein ligase activity involved in mitotic cell cycle
GO:0055085	0.000	110	348	transmembrane transport
GO:0071779	0.000	32	73	G1/S transition checkpoint
GO:0051437	0.000	30	67	positive regulation of ubiquitin-protein ligase activity involved in mitotic cell cycle
GO:0016192	0.000	162	551	vesicle-mediated transport
GO:0009068	0.000	8	9	aspartate family amino acid catabolic process
GO:0006520	0.000	67	198	cellular amino acid metabolic process
GO:0051352	0.000	29	66	negative regulation of ligase activity
GO:0044248	0.000	318	1192	cellular catabolic process
GO:0006921	0.000	24	51	cellular component disassembly involved in execution phase of apoptosis
GO:0046466	0.000	11	16	membrane lipid catabolic process
GO:0032508	0.000	16	29	DNA duplex unwinding
GO:0019885	0.000	6	6	antigen processing and presentation of endogenous peptide antigen via MHC class I
GO:0019637	0.000	206	742	organophosphate metabolic process
GO:0006364	0.000	38	98	rRNA processing
GO:0006508	0.000	162	567	proteolysis
GO:0071702	0.000	328	1248	organic substance transport
GO:0007155	0.000	105	344	cell adhesion
GO:0046496	0.000	16	30	nicotinamide nucleotide metabolic process
GO:0031145	0.000	31	76	anaphase-promoting complex-dependent proteasomal ubiquitin-dependent protein catabolic process
GO:0033554	0.000	246	913	cellular response to stress
GO:2000045	0.000	34	86	regulation of G1/S transition of mitotic cell cycle
GO:0006007	0.000	24	54	glucose catabolic process
GO:0044703	0.000	147	510	multi-organism reproductive process
GO:0043648	0.000	21	45	dicarboxylic acid metabolic process
GO:0007093	0.000	45	124	mitotic cell cycle checkpoint
GO:0051351	0.000	31	77	positive regulation of ligase activity
GO:0006084	0.000	12	20	acetyl-CoA metabolic process
GO:0006928	0.000	163	577	cellular component movement
GO:0071616	0.000	16	31	acyl-CoA biosynthetic process
GO:0044106	0.000	40	108	cellular amine metabolic process
GO:0072331	0.000	39	105	signal transduction by p53 class mediator
GO:0043555	0.000	9	13	regulation of translation in response to stress
GO:0048205	0.000	9	13	COPI coating of Golgi vesicle
GO:0044724	0.000	35	92	single-organism carbohydrate catabolic process
GO:0006629	0.000	179	646	lipid metabolic process
GO:0016043	0.000	642	2612	cellular component organization
GO:0009062	0.001	21	47	fatty acid catabolic process
GO:0006284	0.001	17	35	base-excision repair
GO:0032057	0.001	5	5	negative regulation of translational initiation in response to stress
GO:0051156	0.001	5	5	glucose 6-phosphate metabolic process
GO:0072676	0.001	10	16	lymphocyte migration
GO:0006188	0.001	7	9	IMP biosynthetic process
GO:0051028	0.001	36	97	mRNA transport
GO:0019883	0.001	6	7	antigen processing and presentation of endogenous antigen
GO:0000278	0.001	179	651	mitotic cell cycle
GO:0006066	0.001	56	169	alcohol metabolic process
GO:0031532	0.001	15	30	actin cytoskeleton reorganization
GO:0002576	0.001	17	36	platelet degranulation
GO:0007159	0.001	11	19	leukocyte cell-cell adhesion
GO:1901135	0.001	209	778	carbohydrate derivative metabolic process
GO:0046514	0.001	9	14	ceramide catabolic process
GO:0048194	0.001	9	14	Golgi vesicle budding
GO:0006955	0.001	153	549	immune response
GO:0006090	0.001	12	22	pyruvate metabolic process

Supplementary Table 5: GO-terms (Biological Process) significantly enriched with genes with the highest translational efficiency in C7H2 cells. 2078 genes were tested against the background gene set of 10344 genes. GO-terms with a single associated gene were excluded.

	Pvalue	Count	Size	Term
GO:0032376	0.000	6	7	positive regulation of cholesterol transport
GO:0045071	0.001	11	20	negative regulation of viral genome replication

Supplementary Table 6: GO-terms (Biological Process) significantly enriched with genes with high translational efficiency in C7H2 cells. 2081 genes were tested against the background gene set of 10344 genes. GO-terms with a single associated gene were excluded.

	Pvalue	Count	Size	Term
GO:0044060	0.000	6	6	regulation of endocrine process
GO:0042053	0.000	5	5	regulation of dopamine metabolic process

GO:0060986	0.000	6	7	endocrine hormone secretion

Supplementary Table 7: GO-terms (Biological Process) significantly enriched with genes with medium translational efficiency in C7H2 cells. 2082 genes were tested against the background gene set of 10344 genes. GO-terms with a single associated gene were excluded.

	Pvalue	Count	Size	Term
GO:0042130	0.001	9	16	negative regulation of T cell proliferation

Supplementary Table 8: GO-terms (Biological Process) significantly enriched with genes with low translational efficiency in C7H2 cells. 2080 genes have been tested against the background gene set of 10334 genes. GO-terms with a single associated gene were excluded.

	Pvalue	Count	Size	Term
GO:0019083	0.000	51	130	viral transcription
GO:0006415	0.000	35	78	translational termination
GO:0006614	0.000	36	92	SRP-dependent cotranslational protein targeting to membrane
GO:0072599	0.000	36	94	establishment of protein localization to endoplasmic reticulum
GO:0006414	0.000	35	91	translational elongation
GO:0043241	0.000	43	121	protein complex disassembly
GO:0019058	0.000	59	187	viral infectious cycle
GO:0006402	0.000	53	163	mRNA catabolic process
GO:0006413	0.000	44	132	translational initiation
GO:0006612	0.000	43	129	protein targeting to membrane
GO:0000184	0.000	35	102	nuclear-transcribed mRNA catabolic process, nonsense-mediated decay
GO:0044267	0.000	488	2303	cellular protein metabolic process
GO:0033365	0.000	103	398	protein localization to organelle
GO:0044265	0.000	143	590	cellular macromolecule catabolic process
GO:0006355	0.000	388	1817	regulation of transcription, DNA-dependent
GO:0010556	0.001	431	2042	regulation of macromolecule biosynthetic process
GO:0051252	0.001	399	1882	regulation of RNA metabolic process
GO:0032091	0.001	11	22	negative regulation of protein binding

Supplementary Table 9: GO-terms (Biological Process) significantly enriched with genes with the lowest translational efficiency in C7H2 cells. 2101 genes have been tested against the background gene set of 10344 genes. GO-terms with a single associated gene were excluded.

Enriched processes in NALM6 cells

	Pvalue	Count	Size	Term
GO:0000398	0.000	88	191	mRNA splicing, via spliceosome
GO:0000375	0.000	88	195	RNA splicing, via transesterification reactions
GO:0043436	0.000	207	624	oxoacid metabolic process
GO:0006520	0.000	115	310	cellular amino acid metabolic process
GO:0016071	0.000	176	534	mRNA metabolic process
GO:0006396	0.000	182	566	RNA processing
GO:0051276	0.000	182	574	chromosome organization
GO:0016568	0.000	126	379	chromatin modification
GO:0044281	0.000	399	1483	small molecule metabolic process
GO:0016043	0.000	709	2800	cellular component organization
GO:1901606	0.000	26	50	alpha-amino acid catabolic process
GO:0071704	0.000	1351	5760	organic substance metabolic process
GO:0022613	0.000	76	213	ribonucleoprotein complex biogenesis
GO:0046395	0.000	51	129	carboxylic acid catabolic process
GO:1901607	0.000	21	38	alpha-amino acid biosynthetic process
GO:0002474	0.000	34	76	antigen processing and presentation of peptide antigen via MHC class I
GO:0044282	0.000	60	162	small molecule catabolic process
GO:0002478	0.000	29	62	antigen processing and presentation of exogenous peptide antigen
GO:0000084	0.000	50	129	S phase of mitotic cell cycle
GO:0050878	0.000	110	345	regulation of body fluid levels
GO:0044711	0.000	94	286	single-organism biosynthetic process
GO:0007016	0.000	7	7	cytoskeletal anchoring at plasma membrane
GO:0008214	0.000	12	17	protein dealkylation
GO:0016577	0.000	11	15	histone demethylation
GO:0002479	0.000	28	61	antigen processing and presentation of exogenous peptide antigen via MHC class I, TAP-dependent

GO:0016125	0.000	35	83	sterol metabolic process
GO:0043085	0.000	172	592	positive regulation of catalytic activity
GO:0016192	0.000	174	601	vesicle-mediated transport
GO:0016570	0.000	76	227	histone modification
GO:0019886	0.000	30	69	antigen processing and presentation of exogenous peptide antigen via MHC class II
GO:0044248	0.000	332	1248	cellular catabolic process
GO:0000278	0.000	182	636	mitotic cell cycle
GO:0006807	0.000	980	4056	nitrogen compound metabolic process
GO:0051325	0.000	106	341	interphase
GO:0007596	0.000	94	296	blood coagulation
GO:1901615	0.000	71	211	organic hydroxy compound metabolic process
GO:0000216	0.000	31	73	M/G1 transition of mitotic cell cycle
GO:0006695	0.000	18	34	cholesterol biosynthetic process
GO:0048199	0.000	15	26	vesicle targeting, to, from or within Golgi
GO:0051291	0.000	22	46	protein heterooligomerization
GO:0019885	0.000	6	6	antigen processing and presentation of endogenous peptide antigen via MHC class I
GO:0009084	0.000	10	14	glutamine family amino acid biosynthetic process
GO:0002504	0.000	30	71	antigen processing and presentation of peptide or polysaccharide antigen via MHC class II
GO:0032259	0.000	43	114	methylation
GO:0006103	0.000	9	12	2-oxoglutarate metabolic process
GO:0009068	0.000	7	8	aspartate family amino acid catabolic process
GO:0019883	0.000	7	8	antigen processing and presentation of endogenous antigen
GO:0071826	0.000	38	99	ribonucleoprotein complex subunit organization
GO:0044403	0.000	107	352	symbiosis, encompassing mutualism through parasitism
GO:0032270	0.000	156	544	positive regulation of cellular protein metabolic process
GO:0019048	0.000	97	314	virus-host interaction
GO:0046394	0.000	61	180	carboxylic acid biosynthetic process
GO:0048524	0.000	29	70	positive regulation of viral reproduction
GO:0006479	0.000	29	71	protein methylation
GO:0019221	0.000	69	212	cytokine-mediated signaling pathway
GO:0006139	0.000	879	3640	nucleobase-containing compound metabolic process
GO:0006901	0.000	17	34	vesicle coating
GO:0006732	0.000	51	147	coenzyme metabolic process
GO:0006890	0.000	13	23	retrograde vesicle-mediated transport, Golgi to ER
GO:0043555	0.000	9	13	regulation of translation in response to stress
GO:0048205	0.000	9	13	COPI coating of Golgi vesicle
GO:0034097	0.000	96	315	response to cytokine stimulus
GO:0044764	0.000	162	575	multi-organism cellular process
GO:0006260	0.000	73	229	DNA replication
GO:0006913	0.000	78	248	nucleocytoplasmic transport
GO:0006984	0.000	34	89	ER-nucleus signaling pathway
GO:0006921	0.000	23	53	cellular component disassembly involved in execution phase of apoptosis
GO:0065003	0.001	200	731	macromolecular complex assembly
GO:0051656	0.001	36	96	establishment of organelle localization
GO:0006271	0.001	16	32	DNA strand elongation involved in DNA replication
GO:0030521	0.001	21	47	androgen receptor signaling pathway
GO:0000244	0.001	5	5	assembly of spliceosomal tri-snRNP
GO:0032057	0.001	5	5	negative regulation of translational initiation in response to stress
GO:0055129	0.001	5	5	L-proline biosynthetic process
GO:0051246	0.001	264	996	regulation of protein metabolic process
GO:0072524	0.001	18	38	pyridine-containing compound metabolic process
GO:0006986	0.001	41	114	response to unfolded protein
GO:0032075	0.001	25	60	positive regulation of nuclease activity
GO:0045860	0.001	82	265	positive regulation of protein kinase activity
GO:0032376	0.001	7	9	positive regulation of cholesterol transport
GO:0008610	0.001	110	374	lipid biosynthetic process
GO:0006560	0.001	6	7	proline metabolic process
GO:0044255	0.001	152	541	cellular lipid metabolic process
GO:0051568	0.001	14	27	histone H3-K4 methylation
GO:0033554	0.001	247	933	cellular response to stress
GO:0046782	0.001	24	58	regulation of viral transcription
GO:0006793	0.001	433	1717	phosphorus metabolic process
GO:0030036	0.001	81	264	actin cytoskeleton organization
GO:0019882	0.001	31	82	antigen processing and presentation
GO:0048194	0.001	9	14	Golgi vesicle budding
GO:0051437	0.001	27	68	positive regulation of ubiquitin-protein ligase activity involved in mitotic cell cycle
GO:0051179	0.001	637	2600	localization
GO:0046165	0.001	32	85	alcohol biosynthetic process
GO:0051649	0.001	323	1252	establishment of localization in cell
GO:0071702	0.001	336	1307	organic substance transport
GO:0031060	0.001	12	22	regulation of histone methylation
GO:0050690	0.001	12	22	regulation of defense response to virus by virus

Supplementary Table 10: GO-terms (Biological Process) significantly enriched with genes with the highest translational efficiency in NALM6 cells. 2245 genes were tested for enrichment against the background gene set of 11096 genes. GO-terms with a single associated gene were excluded.

	Pvalue	Count	Size	Term
GO:0060998	0.000	8	11	regulation of dendritic spine development
GO:0007275	0.000	484	2194	multicellular organismal development
GO:0007399	0.000	230	974	nervous system development
GO:0001704	0.000	18	42	formation of primary germ layer
GO:0007423	0.000	63	219	sensory organ development

Supplementary Table 11: GO-terms (Biological Process) significantly enriched with genes with medium translational efficiency in NALM6 cells. 2238 genes were tested for enrichment against the background gene set of 11096 genes. GO-terms with a single associated gene were excluded.

	Pvalue	Count	Size	Term
GO:0006334	0.000	29	74	nucleosome assembly
GO:0015931	0.000	44	129	nucleobase-containing compound transport
GO:0045071	0.000	12	21	negative regulation of viral genome replication
GO:0050658	0.000	39	112	RNA transport
GO:0018401	0.000	5	5	peptidyl-proline hydroxylation to 4-hydroxy-L-proline
GO:0071526	0.000	5	5	semaphorin-plexin signaling pathway
GO:0006403	0.001	40	118	RNA localization

Supplementary Table 12: GO terms (Biological Process) significantly enriched with genes with high translational efficiency in NALM6 cells. 2269 genes were tested for enrichment against the background gene set of 11096 genes. GO-terms with a single associated gene were excluded.

	Pvalue	Count	Size	Term
GO:0035270	0.000	29	65	endocrine system development
GO:0001568	0.000	80	267	blood vessel development
GO:0003002	0.000	53	160	regionalization
GO:0043583	0.000	35	94	ear development
GO:0001525	0.000	59	189	angiogenesis
GO:0060363	0.000	6	6	cranial suture morphogenesis
GO:0042472	0.000	20	44	inner ear morphogenesis
GO:0021602	0.000	7	8	cranial nerve morphogenesis
GO:0010470	0.000	10	15	regulation of gastrulation
GO:0032501	0.000	625	2938	multicellular organismal process
GO:0007267	0.000	122	477	cell-cell signaling
GO:0032940	0.000	96	362	secretion by cell
GO:0048568	0.000	53	176	embryonic organ development
GO:0030857	0.000	6	7	negative regulation of epithelial cell differentiation
GO:0003008	0.000	180	752	system process
GO:0030154	0.000	333	1498	cell differentiation
GO:2000027	0.000	25	68	regulation of organ morphogenesis
GO:0048732	0.000	46	151	gland development
GO:0051216	0.000	26	72	cartilage development
GO:0030212	0.001	9	15	hyaluronan metabolic process
GO:0048663	0.001	13	27	neuron fate commitment
GO:0009653	0.001	255	1127	anatomical structure morphogenesis
GO:0021675	0.001	11	21	nerve development
GO:0060688	0.001	11	21	regulation of morphogenesis of a branching structure
GO:0019226	0.001	91	350	transmission of nerve impulse
GO:0021536	0.001	15	34	diencephalon development
GO:0072358	0.001	107	424	cardiovascular system development
GO:0007275	0.001	469	2194	multicellular organismal development
GO:0048704	0.001	18	45	embryonic skeletal system morphogenesis
GO:0031638	0.001	6	8	zymogen activation
GO:0051965	0.001	6	8	positive regulation of synapse assembly
GO:0017156	0.001	12	25	calcium ion-dependent exocytosis

Supplementary Table 13: GO-terms (Biological Process) significantly enriched with genes with low translational efficiency in NALM6 cells 2219 genes were tested for enrichment against the background gene set of 11096 genes. GO-terms with a single associated gene were excluded.

	Pvalue	Count	Size	Term
GO:0006415	0.000	59	82	translational termination
GO:0006414	0.000	59	94	translational elongation
GO:0006614	0.000	59	96	SRP-dependent cotranslational protein targeting to membrane

GO:0072599	0.000	59	98	establishment of protein localization to endoplasmic reticulum
GO:0000184	0.000	63	109	nuclear-transcribed mRNA catabolic process, nonsense-mediated decay
GO:0019083	0.000	70	135	viral transcription
GO:0006413	0.000	71	139	translational initiation
GO:0043241	0.000	66	126	protein complex disassembly
GO:0006612	0.000	67	134	protein targeting to membrane
GO:0006402	0.000	78	169	mRNA catabolic process
GO:0019058	0.000	82	193	viral infectious cycle
GO:0022411	0.000	81	213	cellular component disassembly
GO:0044265	0.000	177	600	cellular macromolecule catabolic process
GO:0033365	0.000	117	396	protein localization to organelle
GO:0034655	0.000	160	586	nucleobase-containing compound catabolic process
GO:0070727	0.000	185	739	cellular macromolecule localization
GO:0044267	0.000	522	2395	cellular protein metabolic process
GO:0006886	0.000	140	542	intracellular protein transport
GO:0043933	0.000	219	929	macromolecular complex subunit organization
GO:0051100	0.000	19	45	negative regulation of binding
GO:0045184	0.001	218	951	establishment of protein localization
GO:0016482	0.001	134	553	cytoplasmic transport
GO:0034508	0.001	13	28	centromere complex assembly
GO:0031055	0.001	11	22	chromatin remodeling at centromere

Supplementary Table 14: GO-terms (Biological Process) significantly enriched with genes with the lowest translational efficiency in NALM6 cells. 2257 genes were tested for enrichment against the background gene set of 11096 genes. GO-terms with a single associated gene were excluded.

Analysis of microRNA target genes

miRNA (family) target gene predictions base on Targetscan version 6.2 [1] (target genes have been defined using the file *Predicted_Targets_Info.txt* that contains all predicted conserved targets of conserved miRNA families).

In this analysis we evaluated whether genes with low translatoinal efficiciency harbor similar miRNA target sites. Similar to a GO analysis, we thus performed hypergeometric tests to analyse for significant over-representation of predicted miRNA target sites in the 3' UTR of the 5% or genes with the lowest translational efficieny.

Of the in total 536 genes constituting the 5% of genes with lowest translational efficiency in C7H2 341 are also predicted target of at least one miRNA. These genes have been analysed for enrichment against the background gene set consisting of 6899 genes also predicted to be target of at least one miRNA (out of in total 10318 expressed protein coding genes). For NALM6 cells 363 genes have been tested against 7192 background genes (of in total 577 and 11129 test and background genes, respectively).

An enrichment analysis has also been performed on the 5% of the genes with the highest translational efficiency. Some of the miRNA families below (indicated by ‡) have also been found significant in that analysis and thus most likely represent false positive findings.

	miRNA family	p-value	Count	Size	%	host gene†
1	miR-186	0.00	42	556	7.6	6.9
2	miR-23abc/23b-3p	0.03	48	736	6.5	3.1
3	miR-653	0.04	12	136	8.8	2.0
4	miR-137/137ab	0.04	46	717	6.4	1.9
5	miR-340-5p	0.04	59	955	6.2	2.1
6	miR-99ab/100‡	0.05	5	40	12.5	5.3
7	miR-130ac/301ab/301b/301b-3p/454/721/4295/3666	0.05	40	621	6.4	8.0
8	miR-17/17-5p/20ab/20b-5p/93/106ab/427/518a-3p/519d	0.05	52	841	6.2	10.9
9	miR-410/344de/344b-1-3p	0.05	28	410	6.8	

Supplementary Table 15: miRNA families with a significant over-representation of predicted target sites in the 3' UTR of the 5% of genes with the lowest translational efficiency. C7H2 cell line. Count: number of tested genes being a predicted target of the miRNA, Size: total number of predicted target genes for the miRNA detectable on the microarray, %: percentage of predicted target genes being among the genes with the lowest translational efficiency. †: highest expression of any of the miRNAs host genes detected in the respective cell line. For miRNA host gene prediction see [2]. ‡: target sites for that miRNA are also significantly over-represented in 3' UTRs of genes with the highest translational efficiencies.

	miRNA family	p-value	Count	Size	%	host gene†
1	miR-186	0.00	53	563	9.4	4.5
2	miR-203	0.00	51	567	9.0	
3	miR-590-3p	0.00	68	835	8.1	
4	miR-30abcdef/30abe-5p/384-5p	0.00	71	910	7.8	8.9
5	miR-376abd/376b-3p	0.00	17	133	12.8	
6	miR-93/93a/105/106a/291a-3p/294/295/302abcde/372/373/428/519a/520be/520acd-3p/1378/1420ac	0.00	47	583	8.1	2.9
7	miR-340-5p	0.00	68	929	7.3	9.8
8	miR-181abcd/4262	0.00	60	797	7.5	5.9
9	miR-19ab	0.00	59	798	7.4	4.3
10	miR-653	0.00	16	141	11.3	2.0
11	miR-543	0.00	39	482	8.1	2.0
12	miR-17/17-5p/20ab/20b-5p/93/106ab/427/518a-3p/519d	0.00	60	837	7.2	10.7
13	miR-300/381/539-3p	0.00	45	605	7.4	2.1
14	miR-130ac/301ab/301b/301b-3p/454/721/4295/3666	0.01	45	628	7.2	7.1
15	miR-221/222/222ab/1928	0.01	24	288	8.3	2.3
16	miR-136	0.01	16	169	9.5	2.6
17	miR-26ab/1297/4465	0.01	43	604	7.1	10.0
18	miR-376c/741-5p	0.01	15	157	9.6	
19	miR-134/3118	0.01	12	115	10.4	2.0
20	miR-144	0.01	42	589	7.1	1.8
21	miR-382	0.01	14	145	9.7	2.7
22	miR-101/101ab	0.02	39	555	7.0	7.6
23	miR-142-3p	0.03	21	264	8.0	
24	miR-143/1721/4770‡	0.03	21	264	8.0	2.5
25	miR-200bc/429/548a‡	0.03	49	745	6.6	3.5
26	miR-183	0.03	21	270	7.8	2.5
27	miR-196abc	0.04	16	195	8.2	3.5

28	miR-410/344de/344b-1-3p	0.04	30	431	7.0	
29	miR-27abc/27a-3p	0.05	51	802	6.4	3.2
30	miR-448/448-3p	0.05	31	452	6.9	2.4

Supplementary Table 16: miRNA families with a significant over-representation of predicted target sites in the 3' UTR of the 5% of genes with the lowest translational efficiency. NALM6 cell line. Count: number of tested genes being a predicted target of the miRNA, Size: total number of predicted target genes for the miRNA detectable on the microarray, %: percentage of predicted target genes being among the genes with the lowest translational efficiency. †: highest expression of any of the miRNAs host genes detected in the respective cell line. ‡: target sites for that miRNA are also significantly over-represented in 3' UTRs of genes with the highest translational efficiencies.

Detailed information on some miRNA families

Here we list some more information for some of the miRNA families identified in the analysis above. In addition to the expression of their target genes across all 3 pools we add information about the expression of these miRNAs in the analysed cell lines or the lymphoid tissue. Specifically, we evaluate expression of the mature miRNA in C7H2 cells [2], tissue specific expression according to `www.microrna.org` and information from the literature.

miR-30abcdef/30abe-5p/384-5p

- Host gene: host gene relatively high expressed in C7H2 and NALM6 cells.
- Mature miRNA expression in C7H2 cells [2]: miR-30a-5p, miR-30c, miR-30d, miR-30e-5p high expressed.
- Expression profile from microrna.org: miR-30e high in lymphoid tissue.
- Literature: high in ALL [3].

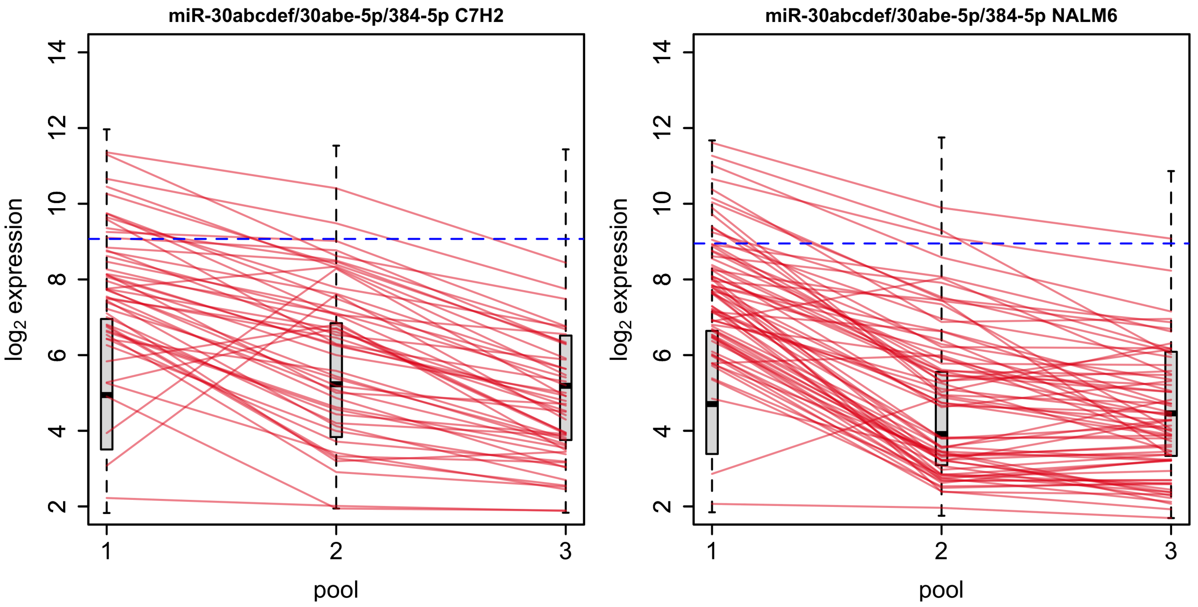

Supplementary Figure 3: Expression of target genes for the miRNA family miR-30abcdef/30abe-5p/384-5p across pools in both cell lines. The boxplot represents expression of all target genes for the miRNA, expression of target genes among the 5% of genes with the lowest translational efficiency are draw in red. The horizontal blue line indicates the (highest) expression of any of the potential host genes for that miRNA in the respective cell line.

miR-93/93a/105/106a/291a-3p/294/295/302abcde/372/373/428/519a/520be/520acd-3p/1378/1420ac

miR-93 is encoded in the miR-106b∼25 cluster, miR-106a in the miR-106a∼363 cluster.

- Host gene: not detectable.
- Mature miRNA expression in C7H2 cells [2]: miR-93, miR-106a very high expressed, miR-105, miR-302abcd, miR-372, miR-373, miR-520be low, not detectable: miR-291a, miR-294, miR-295, miR-302e, miR-428, miR-519a and others.
- Expression profile from microrna.org: miR-93, miR-106a high in lymphoid tissue (B, and T).
- Literature: miR-93, miR-106a high in lymphoid malignancies [3].

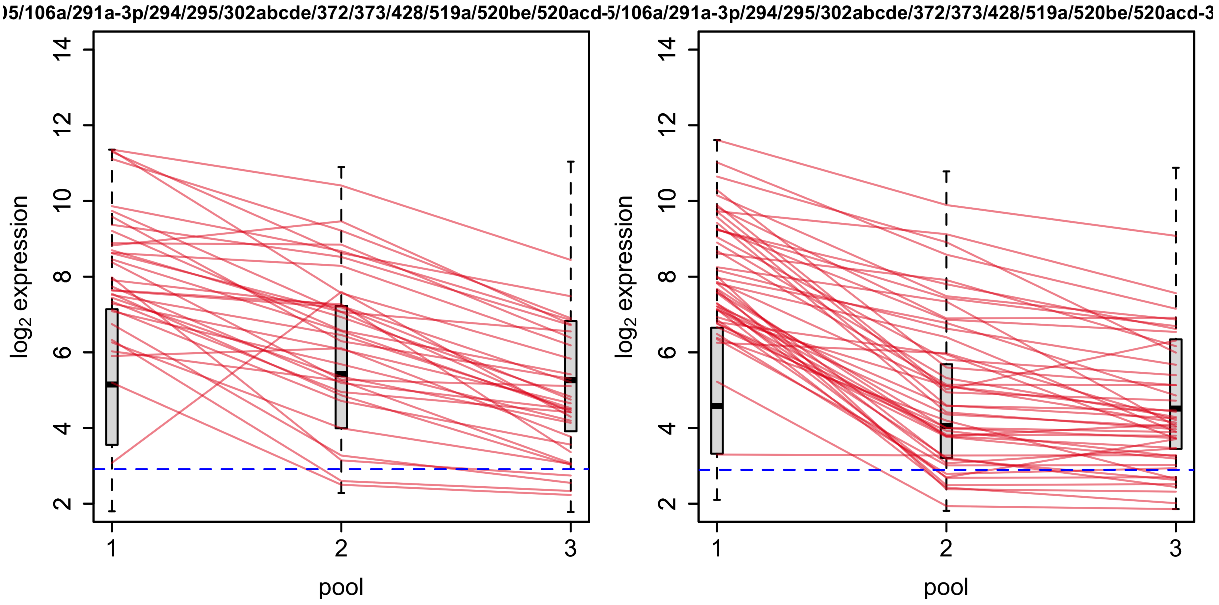

Supplementary Figure 4: Expression of target genes for the miRNA family miR-93/93a/105/106a/291a-3p/294/295/302abcde/372/373/428/519a/520be/520acd-3p/1378/1420ac across pools in both cell lines. The boxplot represents expression of all target genes for the miRNA, expression of target genes among the 5% of genes with the lowest translational efficiency are draw in red. The horizontal blue line indicates the (highest) expression of any of the potential host genes for that miRNA in the respective cell line.

miR-181abcd/4262

miR-181a and miR-181b are both processed from 2 precursors, both clustered together (i.e. miR-181a-1 and miR-181b-1 as well as miR-181a-2 and miR-181b-2). miR-181c and miR-181d are also processed from the same primary transcript.

- Host gene: average expression of one of the host genes.
- Mature miRNA expression in C7H2 cells [2]: miR-181a and miR-181b very high expressed, miR-181c low expressed, miR-181d and miR-4262 not detectable.
- Expression profile from microrna.org: miR-181a, miR-181b high in lymphoid tissue
- Literature: miR-181a, b high in ALL [3].

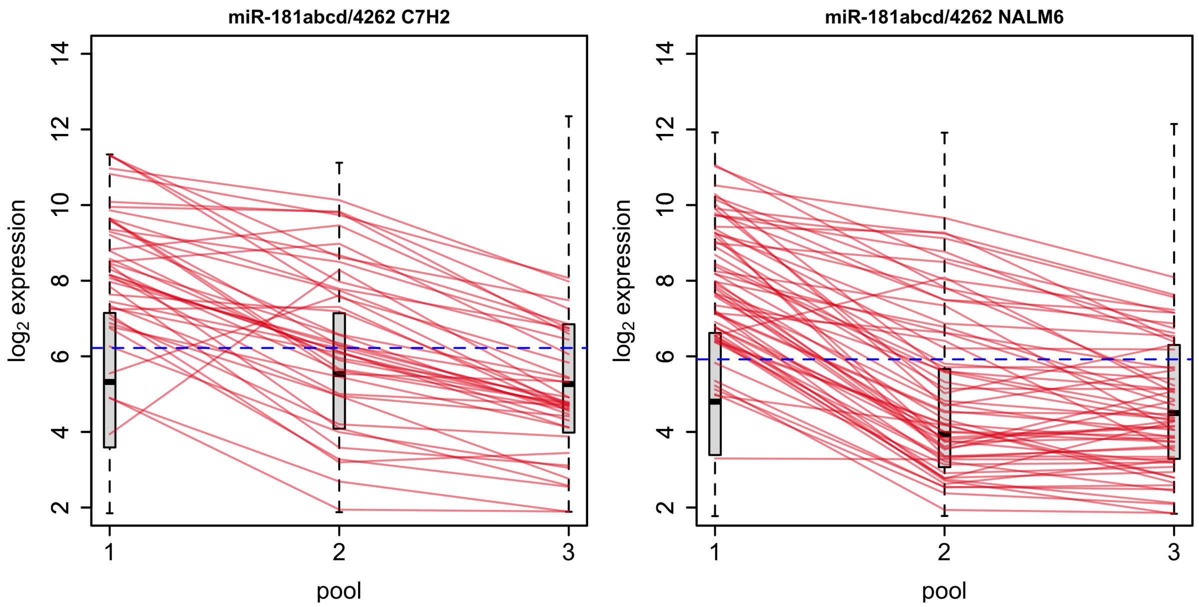

Supplementary Figure 5: Expression of target genes for the miRNA family miR-181abcd/4262 across pools in both cell lines. The boxplot represents expression of all target genes for the miRNA, expression of target genes among the 5% of genes with the lowest translational efficiency are draw in red. The horizontal blue line indicates the (highest) expression of any of the potential host genes for that miRNA in the respective cell line.

miR-19ab

miR-19a and miR-19b-1 are part of the miR-17 92 cluster, miR-19b-2 of the miR-106a 363 cluster.

- Host gene: moderate expression of the host gene in NALM6.
- Mature miRNA expression in C7H2 cells [2]: miR-19a intermediate and miR-19b high expressed.
- Expression profile from microrna.org: high in lymphoma.
- Literature: miR-19a and b from miR-17 92 cluster high in ALL [3].

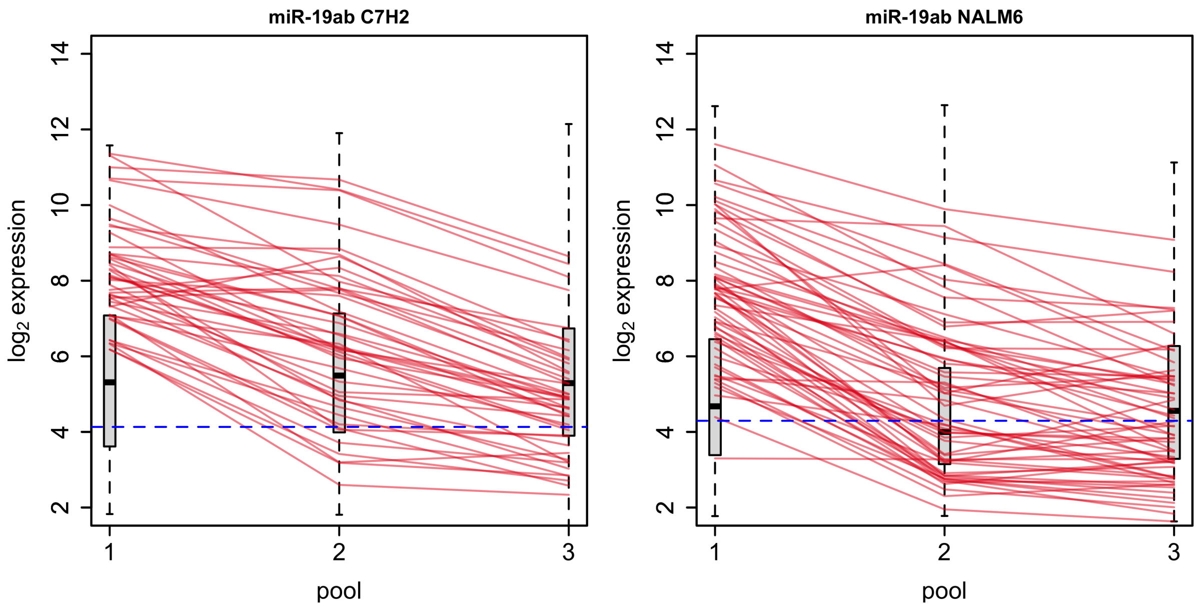

Supplementary Figure 6: Expression of target genes for the miRNA miR-19ab family across pools in both cell lines. The boxplot represents expression of all target genes for the miRNA, expression of target genes among the 5% of genes with the lowest translational efficiency are draw in red. The horizontal blue line indicates the (highest) expression of any of the potential host genes for that miRNA in the respective cell line.

miR-17/17-5p/20ab/20b-5p/93/106ab/427/518a-3p/519d

miR-17 and miR-20a are part of the miR-17 92 cluster, miR-20b and miR-106a of the miR-106a 363 cluster and miR-93 and miR-106b of the miR-106b 25 cluster.

- Host gene: high expression of the host gene in NALM6.
- Mature miRNA expression in C7H2 cells [2]: miR-17-5p, miR-106a very high expressed, miR-20a, miR-93, miR-106b high expressed, not detectable: miR-20b, miR-427 etc.
- Expression profile from microrna.org: miR-17, miR-106a and b, miR-20a, miR-93 high in lymphoid tissue
- Literature: miR-17, part of miR-17 92 cluster overexpressed in ALL [3]. miR-106a, 20b high in lymphoid malignancies. miR-106b, 93 high in lymphoid malignancies.

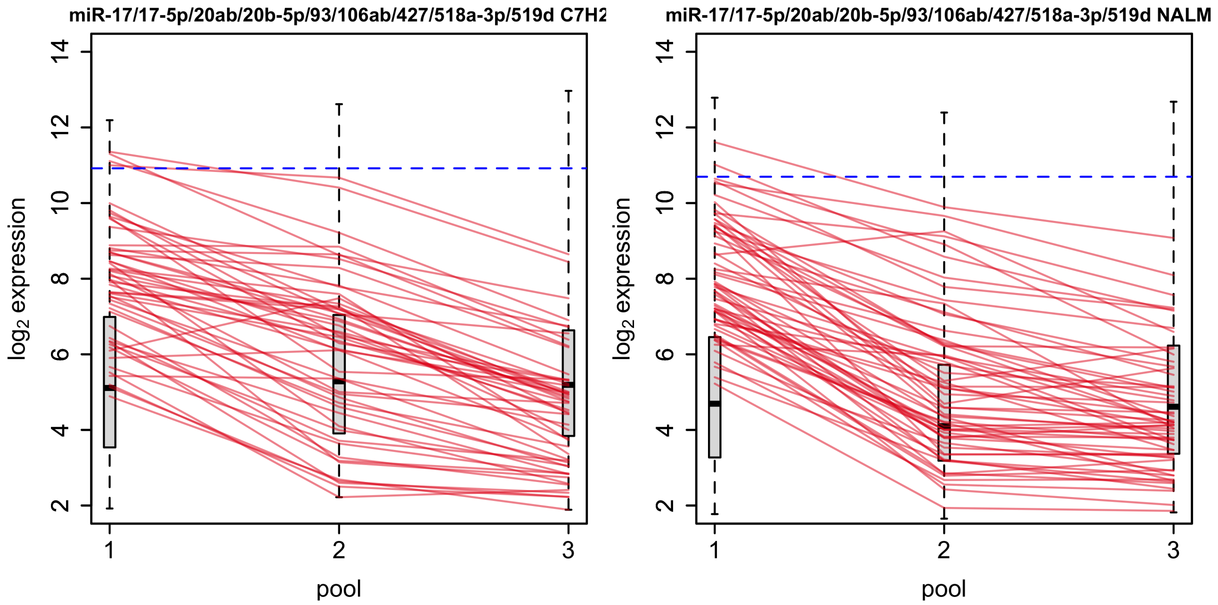

Supplementary Figure 7: Expression of target genes for the miRNA family miR-17/17-5p/20ab/20b-5p/93/106ab/427/518a-3p/519d across pools in both cell lines. The boxplot represents expression of all target genes for the miRNA, expression of target genes among the 5% of genes with the lowest translational efficiency are draw in red. The horizontal blue line indicates the (highest) expression of any of the potential host genes for that miRNA in the respective cell line.

miR-26ab/1297/4465

- Host gene: high expression of the host gene in NALM6.
- Mature miRNA expression in C7H2 cells [2]: miR-26a high expressed, miR-26b low; others not detectable
- Expression profile from microrna.org: miR-26a and b high in lymphoid tissue, specifically B-cells
- Literature: miR-26a high in ALL [3].

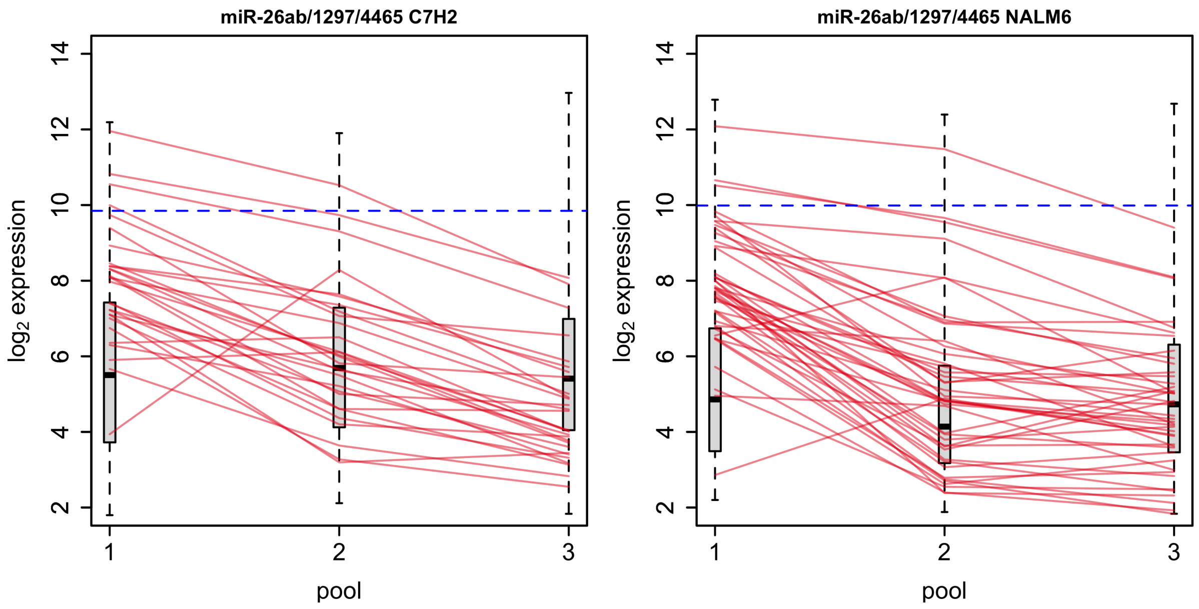

Supplementary Figure 8: Expression of target genes for the miRNA family miR-26ab/1297/4465 across pools in both cell lines. The boxplot represents expression of all target genes for the miRNA, expression of target genes among the 5% of genes with the lowest translational efficiency are draw in red. The horizontal blue line indicates the (highest) expression of any of the potential host genes for that miRNA in the respective cell line.

RNA fractions from sucrose gradient separation

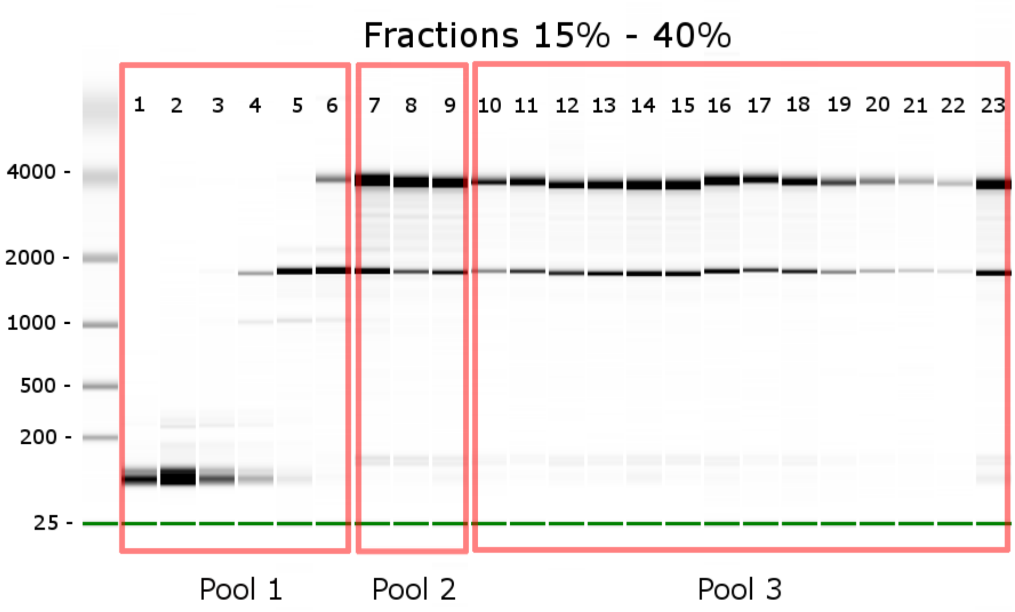

Supplementary Figure 9: Representative example of Agilent gel electrophoresis of RNA fractions obtained by sucrose gradient separation from C7H2 cells. Fractions 1-6 (pool 1) encompass non-ribosome bound RNAs, as suggested by the absence of 28S RNA. Fractions 7-9 form an intermediate pool mostly containing translationally-initiated mRNAs (pool 2). Fractions 10-23 (pool 3) contain mRNAs bound to multiple ribosomes, as evidenced by the presence of ribosomal subunits 18S and 28S.

Bibliography

(1) R. C. Friedman, K. K.-H. Farh, C. B. Burge, and D. P. Bartel, *Genome Research*, Jan. 2009, **19**, 92–105.

(2) J Rainer, C Ploner, S Jesacher, A Ploner, M Eduardoff, M Mansha, M Wasim, R Panzer-Grümayer, Z Trajanoski, H Niederegger, and R Kofler, *Leukemia*, Apr. 2009, **23**, 746–752.

(3) R. V. Sionov, *ISRN hematology*, 2013, **2013**, 348212.
